# Malignant ventricular arrhythmias in patients with chronic myocardial infarction and predictive value of iron-sensitive cardiac magnetic resonance imaging

**DOI:** 10.1186/1532-429X-17-S1-O21

**Published:** 2015-02-03

**Authors:** Ivan Cokic, Avinash Kali, Hsin-Jung Yang, Raymond Yee, Richard Tang, Mourad Tighiouart, Xunzhang Wang, Warren M Jackman, Sumeet S Chugh, James A White, Rohan Dharmakumar

**Affiliations:** 1 Biomedical Sciences - BIRI, Cedars-Sinai Medical Center, Los Angeles, CA, USA; 2 Department of Medicine, Division of Cardiology, London Health Sciences Centre, London, ON, Canada; 3 Biostatistics and Bioinformatics Research Center, Cedars-Sinai Medical Center, Los Angeles, CA, USA; 4 Cedars-Sinai Heart Institute, Cedars-Sinai Medical Center, Los Angeles, CA, USA; 5 Heart Rhythm Institute, University of Oklahoma, Oklahoma City, OK, USA; 6 Stephenson Cardiac Imaging Centre, Dept. of Cardiac Sciences, University of Calgary, Calgary, AB, Canada

## Background

Recent studies in canines have shown that focal iron depositions within chronic scar tissue influences the electrical behavior of infarcted hearts. Further, T2-weighted CMR of post-mortem sudden cardiac death (SCD) victims with chronic myocardial infarction (CMI) have consistently demonstrated regions of signal loss within the CMI territories. To date, the link between the post-infarction iron depositions and malignant ventricular arrhythmias (mVA) in patients with CMI is not known. The aim of this study was to determine the incremental prognostic value of hypointense cores (HIC) identified within CMI using a potentially iron-sensitive CMR approach at 3.0T for the prediction of mVA.

## Methods

A total of 94 CMI patients who underwent routine LGE-CMR protocol at 3.0T prior to ICD implantation for primary and secondary prevention were retrospectively analyzed. Cine steady-state free precession (bSSFP) images (TR/TE=2.6/1.3 ms, flip angle=10^o^, resolution=2x2x6 mm^2^, bandwidth=930 Hz/pixel, and temporal resolution 33ms±5ms) were acquired in short-axis views covering the entire LV. Patients were grouped based on the occurrence of primary endpoint (appropriate ICD therapy, survived cardiac arrest,or SCD), and the presence of HIC within scar on bSSFP CMR. Specifically, we compared the predictive value of HIC for mVAs against other conventional CMR (LVEF, scar size) risk factors of mVA. HIC within CMI on bSSFP as a marker of iron deposition was validated in canine model of CMI.

## Results

**Figure 1:**
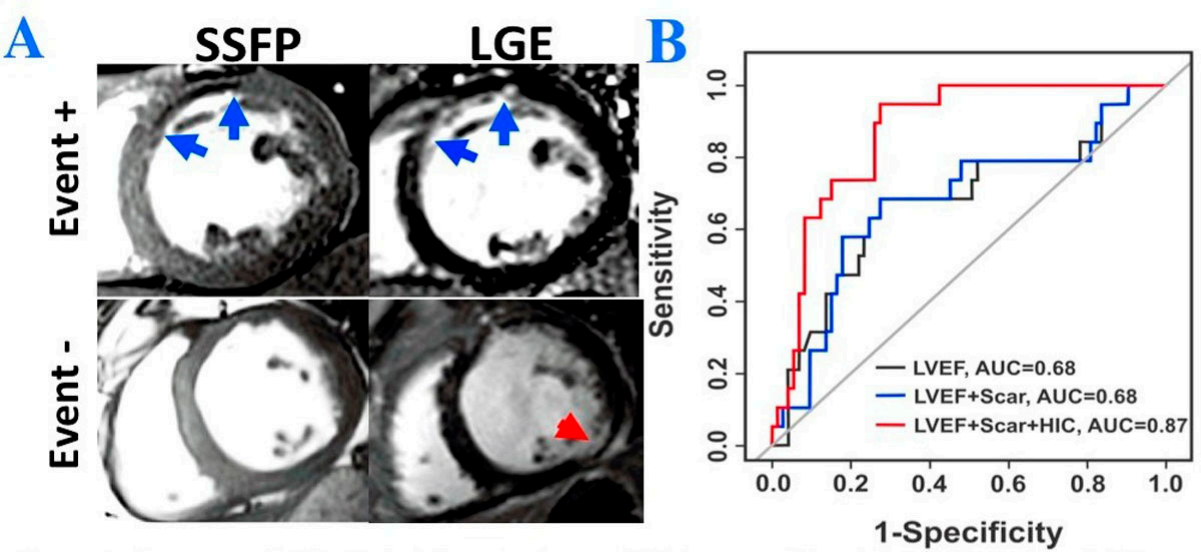
**Presence of HIC within MI territories on SSFP images (A) and Predictive value of HIC on SSFP images for primary endpoint (B). A:** Representatives SSFP and LGE images from two patients receiving ICD therapy; one who met the primary endpoint (Event+) and one who did not meet the primary endpoint (Event-). For the Event+ patient, blue arrows denote that MI region on Late Gadolinium Enhancement (LGE) imaging and the Hypo-Intense Core (HIC) region on SSFP imaging. In the Event- patient, no HIC were observed by SSFP within the MI region, indicated by the red arrow on LGE imaging. **B:** Corresponding ROC curves for LVEF, LVEF + Scar Volume, and LVEF + Scar Volume + HIC for the prediction of the primary endpoint. While the addition of Total Scar Volume alone did not improve the predictive accuracy over LVEF, the addition of HIC improved the AUC from 0.68 to ).87, suggesting additional prognostic value of HIC.

Primary endpoint was met in 19 patients with events occurring 343±269 days after ICD placement. In 19 patients meeting the primary endpoint, 18 were classified as positive for HIC (HIC+) while 1 subject was negative for HIC (HIC-). Among the patients in whom the primary endpoint was not met, there were 28 HIC+ and 47 HIC- patients. ROC analysis demonstrated an additive predictive value of HIC for mVAs (AUC values: 0.68 (LVEF) vs. 0.68 (LVEF + scar volume) vs. 0.87(LVEF + scar volume + HIC)). Histological (validation) studies confirmed that HIC regions in bSSFP images within CMI territories are from iron. Refer to Fig. 1.

## Conclusions

Presence of hypointense core within scar in bSSFP CMR at 3T is a marker of iron deposition within chronic myocardial infarction, which can yield important prognostic information of malignant ventricular arrhythmias over LVEF and scar size.

## Funding

This work was supported in part by:

1. Clinician Scientist Award with the Heart and Stroke Foundation of Ontario, Canada (PI: Dr. JA White).

2. Heart and Stroke Foundation Grant # NA6488 (PI: Dr. JA White).

3. Grant from the National Heart, Lung, and Blood Institute # HL091989 (PI: Dr. R. Dharmakumar).

4. AHA Predoctoral Fellowship # 13PRE17210049 (Mr. Avinas Kali/PI: Dr.R.Dharmakumar).

